# Healthcare-associated adverse events and readmission to the emergency departments within seven days after a first consultation

**DOI:** 10.3389/fpubh.2023.1189939

**Published:** 2023-07-07

**Authors:** Guillaume Gasperini, Leila Bouazzi, Antoine Sanchez, Louis Marotte, Laury Kézachian, Guillaume Bellec, Nicolas Cazes, Maxime Rosetti, Claire Bousquet, Aurélien Renard, Stéphane Sanchez

**Affiliations:** ^1^Emergency Hospital Services, Sainte Anne Army Training Hospital, Toulon, France; ^2^University Committee of Resources for Research in Health (CURRS), University of Reims Champagne-Ardenne, Reims, France; ^3^Ramsay Santé, Hôpital Privé Dijon Bourgogne, Dijon, France; ^4^Medical Educational Institute Les Farfadets, UGECAM PACA-Corse, La Valette-du-Var, France; ^5^Emergency Medical Aid Services, Battalion of Marine Firefighters of Marseille, Marseille, France; ^6^Emergency Hospital Services, Troyes Hospital, Troyes, France; ^7^Public Health and Performance Department, Champagne Sud Hospital, Troyes, France

**Keywords:** healthcare-associated adverse events, emergency departments, emergency medical service, reconsultation, second visit, adverse event

## Abstract

**Introduction:**

The use of emergency hospital service has become increasingly frequent with a rise of approximately 3.6%. in annual emergency department visits. The objective of this study was to describe the reasons for reconsultations to emergency departments and to identify the risk and protective factors of reconsultations linked to healthcare-associated adverse events.

**Materials and methods:**

A retrospective, descriptive, multicenter study was performed in the emergency department of Troyes Hospital and the Sainte Anne Army Training Hospital in Toulon, France from January 1 to December 31, 2019. Patients over 18 years of age who returned to the emergency department for a reconsultation within 7 days were included. Healthcare-associated adverse events in the univariate analysis (*p* < 0.10) were introduced into a multivariate logistic regression model. Model performance was examined using the Hosmer-Lemeshow test and calculated with c-statistic.

**Results:**

Weekend visits and performing radiology examinations were risk factors linked to healthcare associated adverse events. Biological examinations and the opinion of a specialist were protective factors.

**Discussion:**

Numerous studies have reported that a first consultation occurring on a weekend is a reconsultation risk factor for healthcare-associated adverse events, however, performing radiology examinations were subjected to confusion bias. Patients having radiology examinations due to trauma-related pathologies were more apt for a reconsultation.

**Conclusion:**

Our study supports the need for better emergency departments access to biological examinations and specialist second medical opinions. An appropriate patient to doctor ratio in hospital emergency departments may be necessary at all times.

## Introduction

In recent years, the use of **e**mergency hospital **s**ervice (EHS) has become increasingly frequent with a rise of approximately 3.6% in annual emergency department (ED) visits (1, 2). Throughout the world, the EHS seek new ways to improve efficiency in order to accelerate care without any loss of quality (3, 4). To understand the possible outcomes of proposed measures, many risk and protective factors have been studied to highlight any dysfunctions and to provide solutions (5, 6).

In many countries, the rate of a patient returning to the ED a second time in a short period of time, known as a reconsultation, is used as an indicator of the quality of care and safety within the EHS (7–9) since it greatly influences patient flow in its use. Approximately 2% of reconsultations occur within 2–3 days, whereas 7% of visits happen over 7 days, and up to 20% occur over 30 days (10). Healthcare-associated adverse events (AEs) in a reconsultation are the result of a defect in the initial care of a patient. Investigating the underlying causes behind these AEs presents a significant challenge since it seeks to both minimize their occurrence and decrease the number of preventable visits to EHS facilities.

Although the rate of reconsultation is an established measure to indicate the quality of care (7–9), it may be worth questioning its appropriateness for assessing the incidence of healthcare-associated AEs in EDs (11). The objective of this study was to describe the reasons why patients return to the ED within a seven-day timeframe after their first consultation and identify the risk and protective factors of reconsultations linked to healthcare-associated AEs.

## Materials and methods

### Study design

A retrospective, descriptive, multicenter study was conducted in the ED of the Troyes Hospital (*Centre Hospitalier de Troyes*) and the Sainte Anne Army Training Hospital (*Hôpital d’Instruction des Armées*) in Toulon, France from January 1 2019 to December 31, 2019. Troyes Hospital was the only site with a complete technical platform in its ED unlike that of Sainte Anne Army Training Hospital in Toulon which was shared between the two equivalent sites. The latter hospital has fewer beds than the other hospital in the region but it is the only facility for severe trauma, neurovascular emergencies and interventional radiology patients. In the two regions studied, available EHS are limited, and most only accept healthy patients during the day and do not systematically have coronary angiography, neurovascular emergency, neurosurgery and interventional radiology services available.

Even if the ED at Troyes Hospital has greater capacity than that of the Sainte Anne Army Training Hospital, their organization is based on the same standards (12) which include the sorting of patients at reception by a nurse specialized in orienting ED patients (known as the *infirmier d’accueil et d’orientation*) towards two distinct sectors based on the severity of their pathologies. The two sectors are the “short” channel for outpatients only requiring a simple consultation without any examination other than blood tests or X-rays, and a “long” channel into which patients are admitted according to their clinical severity. Patients with serious pathologies are given either individual cubicles or remain in the ED when life-threatening conditions are present. These areas allow for more comprehensive treatment that requires more complex care and continuous monitoring of patient’s vital parameters. Care is administered in the two sites by three senior doctors along with three medical interns. This number of healthcare professionals (HCPs) remains constant 24 h a day, 7 days a week in the ED at Troyes Hospital, but decreases to two doctors and two interns at night (6.30 p.m. to 8:00 a.m.) on weekdays, and on weekends at the Sainte Anne Army Training Hospital.

### Study population

All patients over 18 years of age who had a reconsultation (returned to the ED a second time within 7 days of their first visit) were included. We excluded patients under the age of 18 at the time of the first consultation. In addition, patient files that were created by mistake, duplicated or patients who refused the use of their medical data for research purposes were also excluded.

### Study variables and data collection

The screening and extraction of data and the study of the files were performed using the Résurgences^®^ software. The data were subsequently compiled into a Microsoft Excel. An agreement was set up between the two sites in order to allow a combined analysis in compliance with regulations concerning data privacy. We studied different patient characteristics, patient pathologies and the management of the two consultations. Patient data included gender, age and baseline comorbidities that we measured using the Charlson Comorbidity Index (CCI) (Table 1).

**Table 1:** Baseline comorbidities measured using the Charlson Comorbidity Index (CCI).

Items	Weighting
Myocardial infarction	1 point
Congestive heart failure	1 point
Peripheral vascular diseases	1 point
Cerebrovascular diseases (except hemiplegia)	1 point
Dementia	1 point
Chronic lung diseases	1 point
Connective tissue diseases	1 point
Esophago-gastroduodenal ulcers	1 point
Uncomplicated diabetes	1 point
Mild liver diseases	1 point
Hemiplegia	2 points
Moderate or severe kidney disease	2 points
Diabetes with target organ damage	2 points
Cancer	2 points
Leukemia	2 points
Lymphoma	2 points
Multiple Myeloma	2 points
Moderate to severe liver disease	3 points
Metastasized tumor	6 points
AIDS	6 points

Characteristics of the first visit included how they arrived [by their own means or by medical transport such as an ambulance or an *Véhicule de Secours et d’Assistance aux Victimes* (VSAV)] if they were sent to the ED on the recommendation of a HCP or by a social establishment such as a retirement home or assisted living facility (known as the EHPAD in France), the date and time of the first consultation (working hours from 8:30 a.m. to 6:30 p.m. on weekdays, or during out-of-office time from 6:30 p.m. to 8:30 a.m. on weekdays and/or on a weekend), the elapsed time between administrative registration and discharge from the ED, the seniority of the physician in charge during the first consultation (intern or senior practitioner), the predominant medical specialty for which care was sought, the medical care administered during the consultation [delivery of a treatment, obtaining a medical opinion by a specialist, blood testing and/or medical imaging (X-ray, ultrasound, scanner, or MRI)], the outcome of the consultation including the discharge, standard hospitalization or critical care such as resuscitation or the neurovascular unit (UNV), the severity of the patient’s pathologies, estimated according to a patients classification, known as “Etat patient” (13).

The characteristics relating to the reconsultation were defined by the elapsed time between the two consultations, the patient’s final outcome and the estimated severity according to the patient classification in order to highlight any worsening of the patient’s overall condition. The reasons for the consultation during the second visit were classified according to each category is described in Table 2.

**Table 2:** Reasons for a patient reconsultation in the emergency department of Troyes Hospital and the Sainte Anne Army Training Hospital in Toulon, France in 2019.

Category	Reason for reconsultation	Total patients, *n* (%)
Healthcare-associated AEs	Reconsultation for the same health problem due to an initial diagnosis error, insufficient care or inappropriate treatment prescribed during the first consultation	309 (9.5)
Aggravation	The reason for the second visit is the same and the initial care is correct, but the patient presents an aggravation or a recurrence, foreseeable or not, leading them to consult again	1,232 (37.9)
Mandated visit	The second visit is due to the impossibility of outpatient follow up as planned and accepted by the patient during the first consultation	347 (10.7)
Left without waiting	The patient did not stay until the end of the initial care and returned for the same reason	317 (9.8)
Recall	The second consultation to the ED is planned during the first visit in order to carry out a clinical reassessment, an additional examination or to seek the medical opinion of a specialist	312 (9.6)
Unrelated	The reason for the second consultation has no connection with the first visit and is covered by a different treatment	704 (21.7)
Non-medical	The reason for consultation is not of a medical nature but relates to a social problem (including lack of housing and lack of medical help at home)	25 (0.8)
Total		3,246 (100.0)

A study of patient characteristics based on the reasons cited for the second ED visit was then carried out using the aforementioned criteria. Patients who presented themselves to the ED for non-medical reasons were not studied since no medical care during either consultation was provided. For each patient studied, the reason for reconsultation was determined by two emergency medicine experts. In case of disagreement on the classification, the medical opinion of a third expert was requested after a complete reading of the reports from both consultations.

### Statistical analysis

The continuous variables were described by the mean and the standard deviation in case of normal distribution. Median and the interquartile range were presented in non-standard distribution. The qualitative variables were described as numbers and percentages (%). The continuous variables were compared between the groups using Student’s *t*-test or Mann–Whitney test in the case of a distribution not following the Normal law. Categorical variables were compared using a Chi-square test, or a Fisher’s Exact test in case of expected frequency <5. The risk factors associated with a reason for a reconsultation linked to healthcare-associated AEs in the univariate analysis (*p* < 0.10) were introduced into a multivariate logistic regression model with downward variable selection with a deletion threshold set at 0.05. In order to avoid a possible recruitment bias, the models were adjusted on the center. The odds-ratio (OR) and 95% confidence interval (CI) were obtained from the model. The performance of the models was examined in terms of calibration using the Hosmer-Lemeshow test and calibration by calculating the c-statistic. Statistical tests were performed at the 0.05 significance level. The data was analyzed using SAS software Version 9.4 (SAS Institute, Cary, NC, United States).

## Results

**Figure 1:**
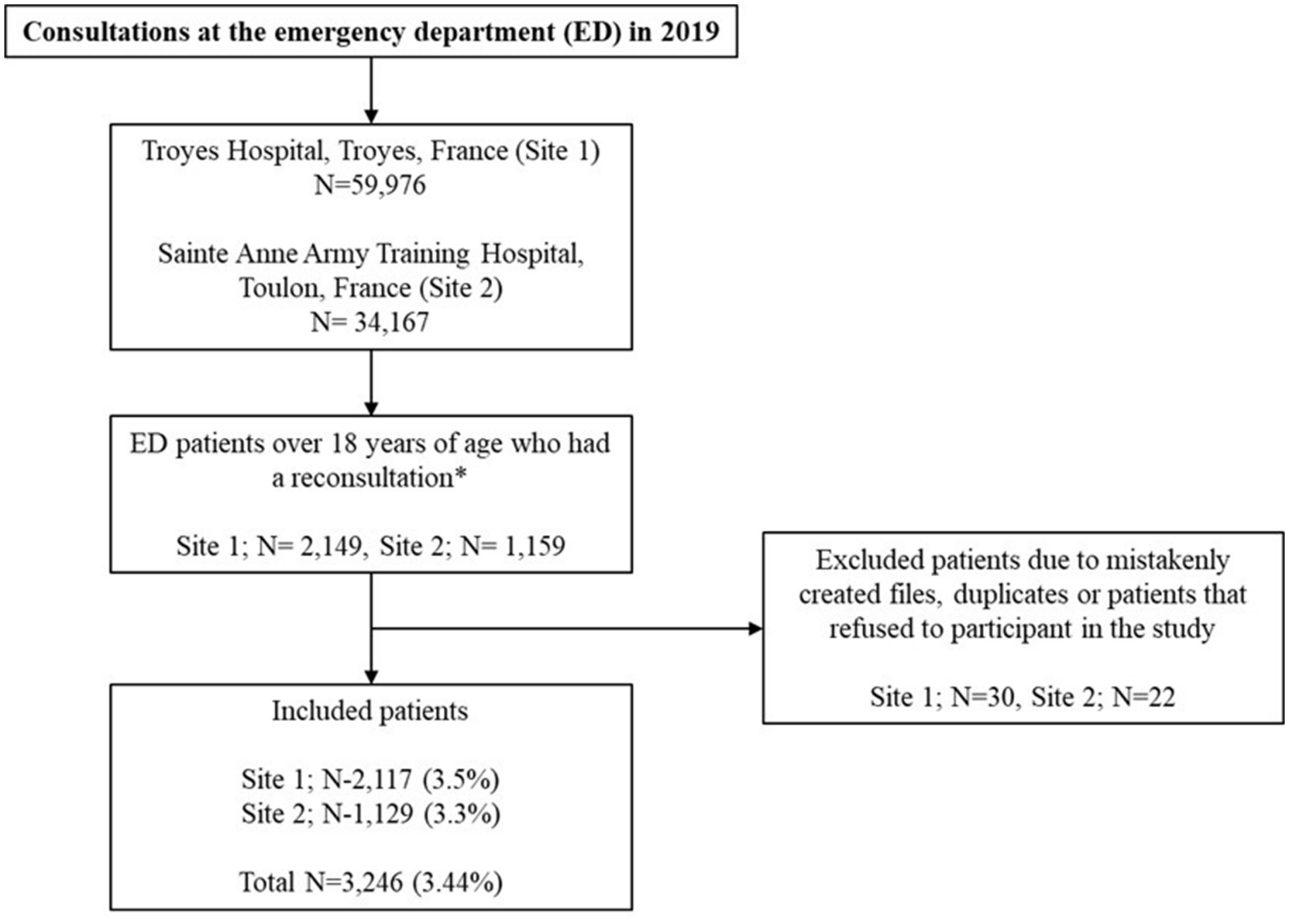
Study flow chart *returned to the emergency department (ED) a second time within 7 days of their first visit.

In 2019, 34,167 ED visits to the Sainte Anne Army Training Hospital and 59,976 visits to Troyes Hospital were made. Of the total amount, 3,246 patients (3.44%) were included in this study (Figure 1). The mean age of patients was 47.7 ± 22.9 years with a male/female ratio of 0.88. There were slightly more first consultations during the day (52.7%) than at night (47.3%). Only 592 (18.2%) consultations were carried out at the advice of an HCP. About one out of two patients (*n* = 1,705, 52.5%) was cared for by an intern during the first consultation (Table 3).

**Table 3:** Characteristics of patients at the first consultation and reconsultation in the emergency department (ED) of Troyes Hospital and the Sainte Anne Army Training Hospital in Toulon, France in 2019.

Characteristics	Patients, *n* (%)
Mean age ± SD, years	47.7 ± 22.9
**Sex**	
Women	1,724 (53.1)
Male	1,522 (46.9)
Consultation on weekends	996 (30.7)
**Consultation period**	
Day	1,712 (52.7)
Night	1,534 (47.3)
Patient referred by a nurse*/physician/other	592 (18.2)
Patients living in the EHPAD	128 (3.9)
Charlson Comorbidity Index (CCI)	0 (0–1)
Support by an intern healthcare professional	1,705 (52.5)
**Severity of the patient pathologies (according to the patient classification)**	
EP1	2,677 (88.8)
EP2	53 (1.8)
EP3	209 (6.9)
EP4	66 (2.2)
EP5	9 (0.3)
Providing a biological examination	1,662 (52.2)
Performing a radiological examination	1,445 (45.4)
Have ongoing treatment	1,948 (61.2)
Asking a specialist for a medical opinion	982 (30.2)
**ED discharge type**	
Residence	2,971 (91.5)
Hospitalization	257 (7.9)
Other	18 (0.5)
Number of days between the first consultation and the second visit, median [IQS]	2 [1 to 4]
Duration of care for the first reconsultation, median [IQS], minutes	196 [90–349]
Total	3,246

SD, standard deviation; EHPAD, accommodation establishment for dependent older aldult; EP, etat patient (patient classification); ED, emergency department; IQS, interquartile range. *specialized in orienting ED patients known as the infirmier d’accueil et d’orientation.

Second ED visits were mainly due to a worsening of the initial pathology (37.9%), while only 309 (9.5%) were the consequence of healthcare-associated AEs (Table 2). The univariate analysis allowed us to highlight the following risk factors for reconsultation linked to healthcare-associated AEs: a first consultation on the weekend (day or night) (*p* = 0.02), patient seen by an intern during the first consultation (*p* = 0.02), and performing a radiology examination (*p* < 0.0001).

Being referred by a HCP, of using biological examinations or getting the medical opinion of a specialist during the first consultation were protective factors. The multivariate analysis carried out (Table 4) confirmed these results by showing that a second ED visit was linked to a healthcare-associated AE stemming from a first consultation occurring on a weekend (*p* < 0.0001), performing a radiology examination during the first visit (*p* < 0.0001) and the absence of biological examinations (*p* < 0.0001) or the medical opinion of a specialist (*p* < 0.0001) during the first visit. Traumatology patients represented the highest number of patients returning to the ED due to healthcare-associated AEs (*n* = 115, 46.4%) (Table 5).

**Table 4:** Risk factors for reconsultation linked to healthcare associated adverse events (AEs) using the multivariate analysis.

	Adjusted OR [95% CI]	*p*-value
First consultation on the weekend	1.32 [1.02–1.17]	0.03
Absence of a biological examination during the first consultation	2.61 [1.99–3.41]	<0.0001
Performing a radiology examination during the first consultation	2.97 [1.99–3.41]	<0.0001
Absence of a specialist medical opinion during the first consultation	1.89 [1.39–2.57]	<0.0001
C-statistics	0.71 [0.68–0.75]	
Quality of fit	0.09	

CI, confidence interval; OR, odds-ratio.

**Table 5:** Predominant medical specialties of disease of patients that went for a second visit to the emergency department within 7 days.

Medical specialty	*n* (%)
Cardiology	12 (4.8)
Visceral surgery	13 (5.2)
Orthopedic surgery	1 (0.4)
Dermatology	6 (2.4)
Endocrinology	1 (0.4)
Gastroenterology	20 (8.1)
Gynecology	9 (3.6)
Geriatrics	4 (1.6)
Neurology	12 (4.8)
ENT specialist	11 (4.4)
Ophthalmology	6 (2.4)
Pulmonology	14 (5.6)
Psychiatrist	3 (1.2)
Rheumatology	10 (4.0)
Stomatology	1 (0.4)
Traumatology	115 (46.4)
Urology	9 (3.6)
Vascular	1 (0.4)
Total	248 (100.0)

## Discussion

Our study shows that most patients returning after 7 days of their first ED visit did so because of the worsening of their initial pathology. The factors for return visits linked to healthcare-associated AEs were that the first consultation occurred on a weekend and/or the results of the radiology examination during the first consultation. In contrast, a biological examination and receiving the medical opinion of a specialist during the first consultation made it possible to limit the number of repeat visits that were linked to a healthcare-associated AE.

We chose to conduct this study in 2019 in order to prevent any possible recruitment bias related to the COVID pandemic. Thus, the population studied was similar between the two sites and the characteristics as well as the rate of reconsultation corresponded to those found in other studies. However, the 9.5% reconsultation rate linked to healthcare-associated AEs was slightly lower than those reported between 12 to 15% in previous literature (14, 15).

Concerning reconsultations as a whole, we observed that the most patients returning to the ED within 7 days since their first visit had mild clinical conditions diagnosed during their first consultation. This is consistent with the data showing that return visits were due to an aggravation of the initial pathology. These expected results have been confirmed both in clinical practice and in other studies (10, 11, 16). It may be worth noting that if the reconsultation rate is not negligible, a reduction to it may seem unlikely in the immediate future due to the unpredictable change in most pathologies that make it difficult for HCPs to identify these patients beforehand. On the other hand, the difficulty of obtaining hospitalization places (2) may force HCPs to use outpatient treatment whenever possible. In order to prevent this type of reconsultations, certain practices could be put in place, such as the 48-h callback of the most vulnerable patients and/or their referral to outpatient care channels as suggested in other studies such as Alshahrani et al. (17) which showed that 71.3% of revisitation complaints were based on no improvement of symptoms among physician related causes and 80.5% were recurrence of the same complaint among patient-related causes (17, 18).

Regarding the risk factors for reconsultation linked to healthcare-associated AEs, the weekend effect highlighted echoes many studies (19–21). Some studies did not find this weekend effect, but they did not take traumatology into account, and have been made after a policy of standardization of medical resources between weekdays and weekends was considered (22). Thus, one of the causes of the weekend effect observed in our study could be a lack of medical resources during the study period which raises the question of what the impact of the patient/doctor ratio is on the quality of care provided in our Eds. Palungwachira et al. (23) reported that the hospital admission rate for a reconsultation slightly decreased by 3.4% when emergency medical resident floor coverage was increased (23).

The French Society of Emergency Medicine (SFMU) recommends a ratio of 1.6 patients per hour/per doctor 7 days a week and 24 h a day (24). In our study, this ratio was respected on weekdays, but not on weekends. Indeed, with only two doctors at the Sainte Anne Army Training Hospital and sometimes unfilled slots at the ED of Troyes Hospital (due to the lack of available doctors), the weekend doctor/patient ratio was lower than that of weekdays in both centers studied.

This observation may be reinforced by the fact that, a first consultation at night was not a predictive factor for reconsultations linked to healthcare-associated AEs in our study. Further research that considers all factors linked to healthcare-associated AEs (25) and the care team’s characteristics should be conducted to confirm what causes the weekend effect and could enable the future implementation of suitable corrective measures.

In our study, the protective factors associated with performing radiology examinations may have been debatable because it was subject to confusion bias. However, after obtaining our results, a second reading of the patient data showed that these examinations were mostly to traumatology patients returning to the ED for persistent pain in connection with a fracture not previously detected on an X-ray during their first visit or they presented pain secondary to a non-optimal cast immobilization. The radiology examination in itself, therefore, was probably not a predictive factor for healthcare-associated AEs, but were indicative of a category of the population at risk of suffering a healthcare-associated AE. The fact that most patients who return to the ED for healthcare-associated AEs were trauma patients (Table 5) echoes this observation and may highlight the importance of having a dedicated healthcare system (26) that is based on treatment protocols that include double reading of X-rays and provide early post-emergency consultations in traumatology (27).

The absence of a biological examination or the medical opinion of a specialist were expected risk factors for reconsultation linked healthcare-associated AEs. Biological tests can reveal criteria that may be hidden by a seemingly reassuring clinical diagnosis (28, 29). Obtaining a second medical opinion by a specialist can help to adjust care and, in some cases, correct the initial diagnosis (24, 30). Our study supports the possible need for permanent access to biological examinations and specialist medical opinions in order to ensure the best possible care for patients. It may be worth noting that the challenge of obtaining a second medical opinion of a specialist during out of office hours (24) may also be a contributing factor to the weekend effect.

Regarding the limitations, we note that the rate of patients returning to the ED was probably underestimated. The two hospitals have sites with EDs nearby (less than a 40 min drive by car). Some patients may therefore have chosen to have their reconsultation there even if it was a less efficient and less-equipped ED (31, 32). In the future, a multicenter study concerning all the hospitals in the region could be conducted to consolidate our findings. It may also be worth noting that ED overcrowding as a variable was relevant to the study, however, we were unable to assess it directly since ED overcrowding remains difficult to quantify in real time and the absence of traceability in medical records did not allow us to explore it.

Using the rate of patients returning to the ED as a criterion to determine of quality of care in the ED may be relevant provided that it is associated with a search for risk factors for reconsultations linked to healthcare-associated AEs. Implementing a system to target specific cases associated with healthcare-associated AEs that cause revisits to the ED can save time in identifying necessary corrective measures.

## Conclusion

Healthcare-associated AEs has a significant impact on patient outcomes and the use of healthcare resources. Identifying and addressing the underlying factors responsible for reconsultations such as the weekend effect and difficulty obtaining a specialist medical opinion during out-of-office hours is crucial to improving patient safety and reducing the burden on the EHS. It is also essential to abide by the appropriate patient/doctor ratio at all times in order to prevent the observed weekend effect. By adopting a proactive approach to patient safety and healthcare management, EDs can ensure optimal care delivery while also reducing the incidence of reconsultations.

## Data availability statement

The data analyzed in this study is subject to the following licenses/restrictions: These data are provided especially for the purposes of this study. They are therefore not available to the public. Requests to access these datasets should be directed to SS via stephane.sanchez@hcs-sante.fr.

## Author contributions

GG, SS, and CB were involved in the conception and design of the study. SS, CB, GG, and AR were the coordinator of the study. GG, CB, and MR were responsible for the data collection. GG, AS, and CB wrote the first draft. LB was in charge of the analysis. GG, AR, SS, LM, LK, GB, NC, and AS were involved in the interpretation, critically reviewed the first draft. All authors contributed to the article and approved the submitted version.

## Conflict of interest

The authors declare that the research was conducted in the absence of any commercial or financial relationships that could be construed as a potential conflict of interest.

## Publisher’s note

All claims expressed in this article are solely those of the authors and do not necessarily represent those of their affiliated organizations, or those of the publisher, the editors and the reviewers. Any product that may be evaluated in this article, or claim that may be made by its manufacturer, is not guaranteed or endorsed by the publisher.
